# DNA methylation subtypes guiding prognostic assessment and linking to responses the DNA methyltransferase inhibitor SGI-110 in urothelial carcinoma

**DOI:** 10.1186/s12916-022-02426-w

**Published:** 2022-07-18

**Authors:** Juan Li, Yuan Liang, Jian Fan, Chunru Xu, Bao Guan, Jianye Zhang, Bin Guo, Yue Shi, Ping Wang, Yezhen Tan, Qi Zhang, Changwei Yuan, Yucai Wu, Liqun Zhou, Weimin Ci, Xuesong Li

**Affiliations:** 1 Department of Urology, Peking University First Hospital, Beijing, 100034 China; 2 CAS Key Laboratory of Genomic and Precision Medicine, Beijing Institute of Genomics, Chinese Academy of Sciences and China National Center for Bioinformation, Beijing, 100101 China; 3 University of Chinese Academy of Sciences, Beijing, 100049 China; 4 Institute of Urology, Peking University, Beijing, 100034 China; 5 National Urological Cancer Center, Beijing Key Laboratory of Urogenital Diseases (Male) Molecular Diagnosis and Treatment Center, Beijing, 100034 China; 6 Institute for Stem cell and Regeneration, Chinese Academy of Sciences, Beijing, 100101 China

**Keywords:** Upper tract urothelial carcinoma, Urothelial carcinoma of the bladder, Whole-genome bisulfite sequencing, DNA methylation subtype, Prognosis, DNA methyltransferase inhibitor

## Abstract

**Background:**

At present, the extent and clinical relevance of epigenetic differences between upper tract urothelial carcinoma (UTUC) and urothelial carcinoma of the bladder (UCB) remain largely unknown. Here, we conducted a study to describe the global DNA methylation landscape of UTUC and UCB and to address the prognostic value of DNA methylation subtype and responses to the DNA methyltransferase inhibitor SGI-110 in urothelial carcinoma (UC).

**Methods:**

Using whole-genome bisulfite sequencing (*n* = 49 samples), we analyzed epigenomic features and profiles of UTUC (*n* = 36) and UCB (*n* = 9). Next, we characterized potential links between DNA methylation, gene expression (*n* = 9 samples), and clinical outcomes. Then, we integrated an independent UTUC cohort (Fujii et al., *n* = 86) and UCB cohort (TCGA, *n* = 411) to validate the prognostic significance. Furthermore, we performed an integrative analysis of genome-wide DNA methylation and gene expression in two UC cell lines following transient DNA methyltransferase inhibitor SGI-110 treatment to identify potential epigenetic driver events that contribute to drug efficacy.

**Results:**

We showed that UTUC and UCB have very similar DNA methylation profiles. Unsupervised DNA methylation classification identified two epi-clusters, Methy-High and Methy-Low, associated with distinct muscle-invasive statuses and patient outcomes. Methy-High samples were hypermethylated, immune-infiltrated, and enriched for exhausted T cells, with poor clinical outcome. SGI-110 inhibited the migration and invasion of Methy-High UC cell lines (UMUC-3 and T24) by upregulating multiple antitumor immune pathways.

**Conclusions:**

DNA methylation subtypes pave the way for predicting patient prognosis in UC. Our results provide mechanistic rationale for evaluating SGI-110 in treating UC patients in the clinic.

**Supplementary Information:**

The online version contains supplementary material available at 10.1186/s12916-022-02426-w.

## Background

Urothelial carcinomas (UCs) are the fourth most common tumors, and urothelial carcinoma of the bladder (UCB) accounts for 90–95% of UCs, while upper tract urothelial carcinoma (UTUC) is uncommon and account for 5–10% of UCs [1]. Although genomic studies have revealed significant differences in the prevalence of somatic alterations between UTUC and UCB [2], the extent and clinical relevance of epigenetic differences between UTUC and UCB remain largely unknown.

Increasing evidence suggests that DNA methylation is closely related to tumor progression in UCB. Initially, studies of DNA hypermethylation were focused on CpG islands of potential candidate genes [3]. Unique DNA methylation patterns distinguish noninvasive and invasive UCBs and are associated with clinical outcome [4]. However, DNA methylation profiling in UTUC remains rare. Two recent studies of UTUC have used the Infinium HumanMethylation850 (EPIC) BeadChip array [5, 6]. Neither study assessed the relationship between treatment and prognostic risk subtypes.

Comparative analysis of DNA methylation alterations between UTUC and UCB has been limited, and studies often focus on selected cancer-related genes. It has been shown that promoter methylation is more common and extensive in UTUC than in UCB [7, 8] for a few genes. However, in our previous study, comparison of whole-genome sequencing [9, 10] and whole-genome bisulfite sequencing (WGBS) [11] data of UTUC and UCB in both tumor tissue and urine sediment samples identified that similar genomic CNV and DNA methylation alterations were present in both cancer types, indicating a potential opportunity for similar management strategies for UC. Thus, contrasting of DNA methylation profiling between them would aid the development of diagnostics, prognostication, and even therapeutics for UC.

In this study, we investigated the epigenomic features and profiles of UTUC and UCB. We further characterized DNA methylation subtypes to explore potential risk stratification of tumors. Furthermore, integrative analysis was used to evaluate therapeutic efficacy and targets of the DNA methyltransferase inhibitor SGI-110 in two UC cell lines, T24 and UMUC-3. Our findings support a potential opportunity for similar management strategies for UC, and DNA methylation subtypes guiding prognostic assessment and linking to responses the DNA methyltransferase inhibitor SGI-110 in UC.

## Methods

### Study design and participants

**Fig. 1:**
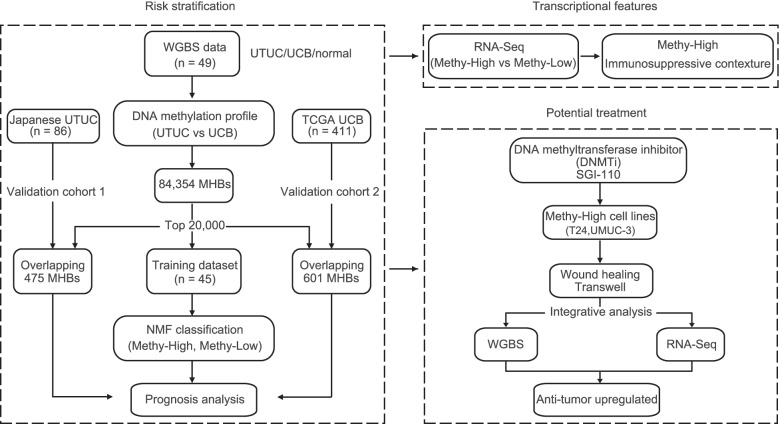
Flow diagram of the study. A training cohort of 45 UC samples and two validation cohorts were used for classification and prognosis analysis. RNA-seq data of 9 UTUC patients revealed the transcriptional features of the two subtypes. WGBS and RNA-seq were performed on SGI-110-treated cell lines

This study includes three parts: (1) risk stratification, (2) transcriptional features, and (3) potential treatment (Fig. 1). Thirty-six fresh-frozen surgically resected primary UTUC tumors and four matched adjacent tissues were collected retrospectively from the Peking University First Hospital. Nine bladder cancer cases were obtained from our previous study [11]. The follow-up time for the patients was 40 months. The study was approved by the Ethics Committee of Peking University First Hospital. Two independent validation cohorts included 86 UTUC patients from Japanese cohort [5] and 411 UCB patients from The Cancer Genome Atlas (TCGA). The follow-up time for Japanese cohort was 200 months. The follow-up time for TCGA cohort was 5000 days.

### Laser Capture Microdissection (LCM)

Firstly, matched adjacent tissues were embedded using OCT (SAKURA, #4583). Then, the frozen sections were stained with hematoxylin-eosin to confirm normal morphology. Finally, adjacent normal urothelium on the mmi membrane slide (MMI, #50103) was collected by CCP+IX83/CellCut Plus microsystem (Leica, CM1950).

### Whole-genome bisulfite sequencing

For whole-genome sequencing, genomic DNA from fresh cancer tissue samples was isolated using the QIAamp DNA Mini Kit (QIAGEN#51304). The DNA sequencing libraries were prepared using the NEB Next Ultra II DNA Library Prep Kit for Illumina (New England Biolabs, MA, US) following the manufacturer’s instructions. Firstly, Covaris S2 Ultrasonicator instrument (Covaris Inc., MA, US) was used to fragment the genomic DNA. Then, DNA fragment was repaired and 3′ dA-tailed using the NEB Next Ultra II End Repair/dA-Tailing Module (NEB, #E7546 L). NEB Next Ultra II Ligation Module (NEB, #E7595 L) was used to ligate paired-end adaptors. After purification by AMPure XP beads (Beckman, #A63881), CT transformation was required for the product through EZ DNA Methylation-Gold Kit™ (Zymo, #D5006). Further PCR amplification required 8–10 cycles, the DNA library was purified by AMPure XP beads again. The quality of DNA library and sequencing were determined by Nova Seq 6000, according to the manufacturer’s instructions, generating 2 × 150-bp paired-end reads.

For data preprocessing, first of all, the quality of short DNA reads was controlled by Trimmomatic (version 0.36) with default parameters. Then, hg19 reference genome (http://genome.ucsc.edu/) was downloaded from UCSC. The 48502 bp Lambda genome was also included in the reference sequence for calculating bisulfite conversion rate. Paired-end BS-seq reads were mapped against the reference by Bismark (version 0.16.3) with parameters: -N 1 -X 600. Bismark (version 0.16.3) was used to remove PCR duplication. BamUtil (version 1.0.14) was used to examine whether pair-end reads overlapped and the overlapped part was trimmed from one end to prevent counting twice from the same observation. Finally, clean data was obtained for subsequent analysis.

### Cell lines and in vitro drug treatments

T24 and UMUC-3 cell lines were cultured in DMEM (HyClone), 10% fetal bovine serum (BI), and 1% Penicillin-Streptomycin (Life). Cell lines were treated with SGI-110 (Selleck, #S7013) at concentrations of 1, 2, and 4 μM.

### Transwell assay

Transwell chambers (Corning, #3422) in 24-well plates were used for in vitro cell migration and invasion detection. In brief, 500 μL of 10% FBS-containing medium was pre-added to the 8 μM lower chambers. UMUC-3 cells were resuspended in medium free of FBS and 200 μL of the cell suspension containing 1 × 10^4^cells was added to the upper chamber, and cultured at 37 °C with 5% CO_2_ for 16.5 h. T24 cells were cultured for 20.5 h by same procedure. After discarding medium, both cell lines were fixed with 4% methanol for 2 h, and stained with 0.1% crystal violet; and then were photographed under an inverted microscope in five randomly selected fields of view, with three replicates for each specimen. Finally, we counted the number of cells that passed through and got the average of the 5 fields.

### Wound healing assay

Each well was seeded with 25,000 cells in good condition in a 96-well plate. Until the cell density was raised above 90%, monolayer cells were scratched by a straight line using a sterile 96-well pin block, which can mimic an incision wound. After that, PBS was utilized to wash cells for removing cell debris. Then, fresh cell culture medium at 37 °C was added into a 96-well plate. Cultured and the photographs of the scratch wound were obtained every 2 h using IncuCyte (Essen BioScience, Inc., USA). The wound confluence of scratch area measured to reflect the speed of cell migration by IncuCyte.

### RNA sequencing (RNA-seq) and differential gene expression analysis

Total RNA was extracted using TRIzol reagents from samples, and total RNA was isolated with a Ribo-off rRNA Depletion Kit (Vazyme, #N406). Library construction was conducted using the VAHTS Universal V8 RNA-seq Library Kit for Illumina (Vazyme, #NR605) according to the manufacturer’s instructions. Quality control and sequencing were performed by Nova Seq 6000. The quality of short DNA reads was controlled by Trimmomatic with default parameters (version 0.36). The good quality PE reads were aligned with the human reference genome hg19 using the hisat2 software (version 2.0.5). Mapped reads of high quality were picked by Samtools (q>20). The uniquely mapped reads were counted using the HTSeq python package (version 0.9.1). Transcripts per million (TPM) was calculated by StringTie (version 1.3.5). Differentially expressed genes between Methy-high and Methy-low subtypes were determined using the R package DEseq2 software (fold-change cut-off = 2.0, *p* value cut-off = 0.05). Differentially expressed genes between treatment and no treatment cell lines were determined by GFOLD software with default parameters (GFOLD cut-off = 0.3).

### Methylation analysis at the 10-kb bin level

For 10-kb bin analysis, hg19 genome was divided into 288,113 bins of 10k bases. Then the number of “C” bases and “T” bases were counted as methylated (denoted as MC) and unmethylated (denoted as MT), respectively, in each bin. Then, 5mC% is estimated as MC/ (MC + MT).

### Methylation analysis at the block level

MHB (methylation haplotype block) is the block of tightly coupled CpG sites in the human genome. Biologically, MHBs are the regions of genome that tend to be tightly co-regulated on the epigenetic status. A total of 799,820 MHBs were identified in urothelial carcinoma and normal tissues according to the previous studies [12], which covered 11,526,876 bp of the genome, an average of 137 bp per MHB. In brief, methylation haplotype load (MHL) was a weighted mean of the fraction of fully methylated haplotypes and substrings at different lengths. MHL was calculated to quantify the methylation level of each MHB. We removed MHBs with NA values across all samples and obtained 84,354 MHBs in all samples for further analysis.

### Methylation subtype classification for urothelial carcinoma by NMF

For discovery cohort, we selected the top 20,000 most-varying MHBs by standard deviation of MHL across samples. The R package non-negative matrix factorization (NMF, runs=100, rank=2) was used to deconvolute the methylation signature. The components (signature contributors) for each class were identified by the extractFeatures () R function. For Japanese cohort and TCGA cohort, due to differences of data, we performed the above NMF method by capturing the average methylation levels of overlapping regions between top 20,000 most-varying MHBs and EPIC array sites.

### Genome distribution of differentially methylated MHBs

The Homer annotatePeaks tool was used to determine the distribution of hyper- and hypomethylated MHBs in the genome. The enrichment ratio (Log2 Ratio (obs/exp) calculated by Homer represents the degree of distribution of elements. Various types of repeat sequences were downloaded from UCSC. The enrichment ratio refers to the observed ratio of hypo MHBs occurred in different evolutionary Alu elements to their proportion.

### Functional enrichment analysis

Gene ontology (GO) analysis of genes was performed using metascape (http://metascape.org) and ClusterProfiler, an R package that analyzed and visualized functional profiles (GO) of genes and gene clusters. Gene set enrichment analysis GSEA (http://software. broadinstitute.org/gsea/index.jsp) was used to assess population phenotype-related pathways, which estimated the significance of a set of genes. Single sample GSEA (ssGSEA) was extended by GESA, which calculated the enrichment score of the given gene set in each sample. GO terms and enriched gene sets with a *P* value < 0.05 were considered statistically significant.

### Tumor microenvironment analysis

The ESTIMATE R package (version 1.0.13) was used to measure infiltrating stromal and immune cells [13]. We used the TPM data to generate stromal score and immune score. Tumor immunophenotype profiling (TIP) is software that resolves tumor immunophenotype profiling [14]. RNA-seq count data was used to resolve the activity score of anticancer immunity across seven steps in the discovery cohort. We downloaded the score of TCGA bladder cancers from pancanceranalysis module on the website (http://biocc.hrbmu.edu.cn/TIP). The MCPcounter R package (version 1.2.0) was used to estimate the abundance in immune microenvironment [15].

### Repeat expression analysis

A gene transfer format (GTF) file of the whole-genome repeat element loci marked by RepeatMasker was downloaded from the UCSC genome browser table. The transcription of repetitive elements was calculated using the featureCounts command from the subread tools (version 2.0.1). We summed the read counts of each repeat class, and the percentage of reads for each repeat class was calculated as the ratio of the repeat class count to the total reads in the bam file.

### Statistical analysis

Differences in methylation levels of elements between tissues were tested using ANOVA. Differences in correlation between groups were tested using *t*-tests. Pearson’s correlation coefficients were calculated to evaluate the correlations of pairwise methylation levels. The Kaplan**–**Meier analysis and Cox proportional hazards model analysis were used to evaluate the associations of the subtypes with overall survival and progression-free survival, and *P* value < 0.05 was considered statistically significant. The Wilcoxon rank-sum test was used to assess statistical significance for cell populations of two methylation subtypes. All statistical tests were executed by R 4.0.5.

## Results

### Comparison of DNA methylation profiles of UTUC and UCB

**Fig. 2:**
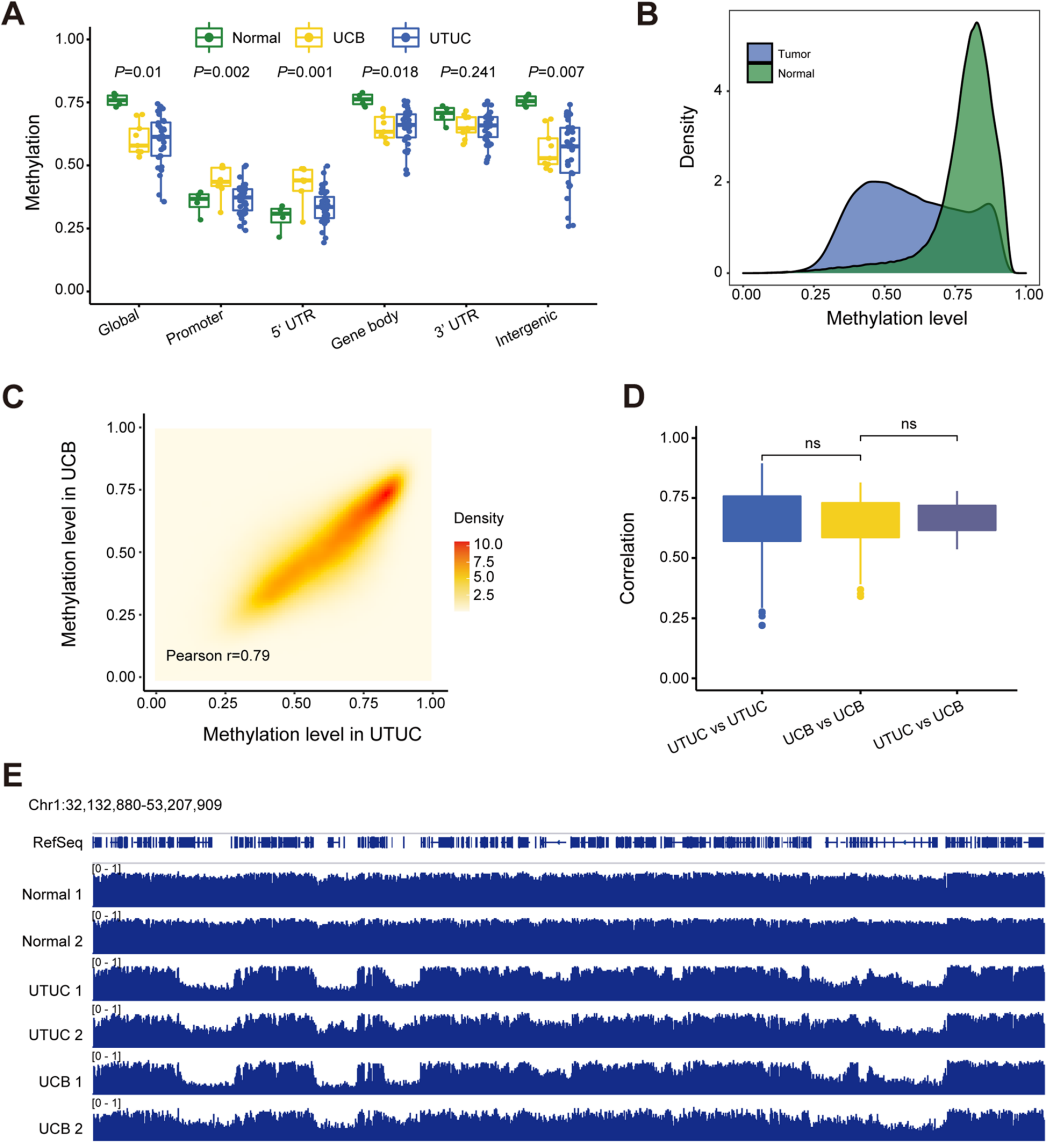
WGBS analysis reveals that the DNA methylation profiles of UTUC and UCB are similar. **A** Global changes in average 5mC levels in different genomic elements determined by WGBS (the promoter is defined as ±1000 bp of the TSS) in UCB (*n* = 9), UTUC (*n* = 36), and paired adjacent urothelium specimens (*n* = 4). **B** Distribution of the average methylation levels of all genome-wide 10-kb bins in paired UTUC tumors and adjacent urothelium (*n* = 4). **C** Density plot of average DNA methylation within all genome-wide 10-kb bins in UCB (*n* = 9) and UTUC (*n* = 36) tumor samples. **D** Box plot of the correlation of the average methylation level within all genome-wide 10-kb bins within and between the two groups. **E** Graphical representation of the dynamic 5mC pattern of a region from chromosome 1. Two normal urothelium samples, UTUC tumor tissues and UCB tumor tissues were randomly selected

To investigate potential differences in the DNA methylation landscape between UTUC and UCB, we detected the genome-wide DNA methylation profiles of primary tumor tissues, including 36 UTUC samples and 9 UCB samples, and 4 adjacent normal tissues (Additional file 1: Fig. S1A) using whole-genome bisulfite sequencing (WGBS) (Additional file 2). As expected, when we compared the two types of tumors to adjacent normal tissues, we found that the DNA methylation level decreased at the genome-wide level and increased in promoter regions and 5′ UTR regions (Fig. 2A and Additional file 1: Fig. S1B). The density plot further showed that the global level of DNA methylation was decreased in UC tumor tissues compared with normal adjacent tissues (Fig. 2B and Additional file 1: Fig. S1D). Strikingly, the landscape of DNA methylation in UTUC and UCB was similar (Fig. 2C, D and Additional file 1: Fig. S1C). As shown in Fig. 2E, for one representative genomic locus, chr1: 32,132,880-53,207,909, global demethylation was evidenced in both UTUC and UCB, and the DNA methylation profiles were very similar between UTUC and UCB.

### DNA methylation subtype classification

Next, we investigated whether there were distinct DNA methylation subtypes within UC. Recently, Guo et al. [12] demonstrated the superior sensitivity of multi-CpG haplotypes in detecting tissue-specific signatures by an exhaustive search of tissue-specific methylation haplotype blocks (MHBs) across the full genome and proposed a block-level metric termed methylated haplotype load (MHL). We identified 84,345 MHBs in UC tumor tissues and normal adjacent tissues using this method. Consistent with the global demethylation, we identified 15,089 hypomethylated MHBs and 362 hypermethylated MHBs in UC tumor tissues compared to normal adjacent tissues (Additional file 1: Fig. S2A and S2B). As expected, the hypomethylated MHBs were significantly enriched in repeat regions, particularly in short interspersed elements (SINEs), of which Alu Y was the main subtype (Additional file 1: Fig. S2C-S2E).

**Fig. 3:**
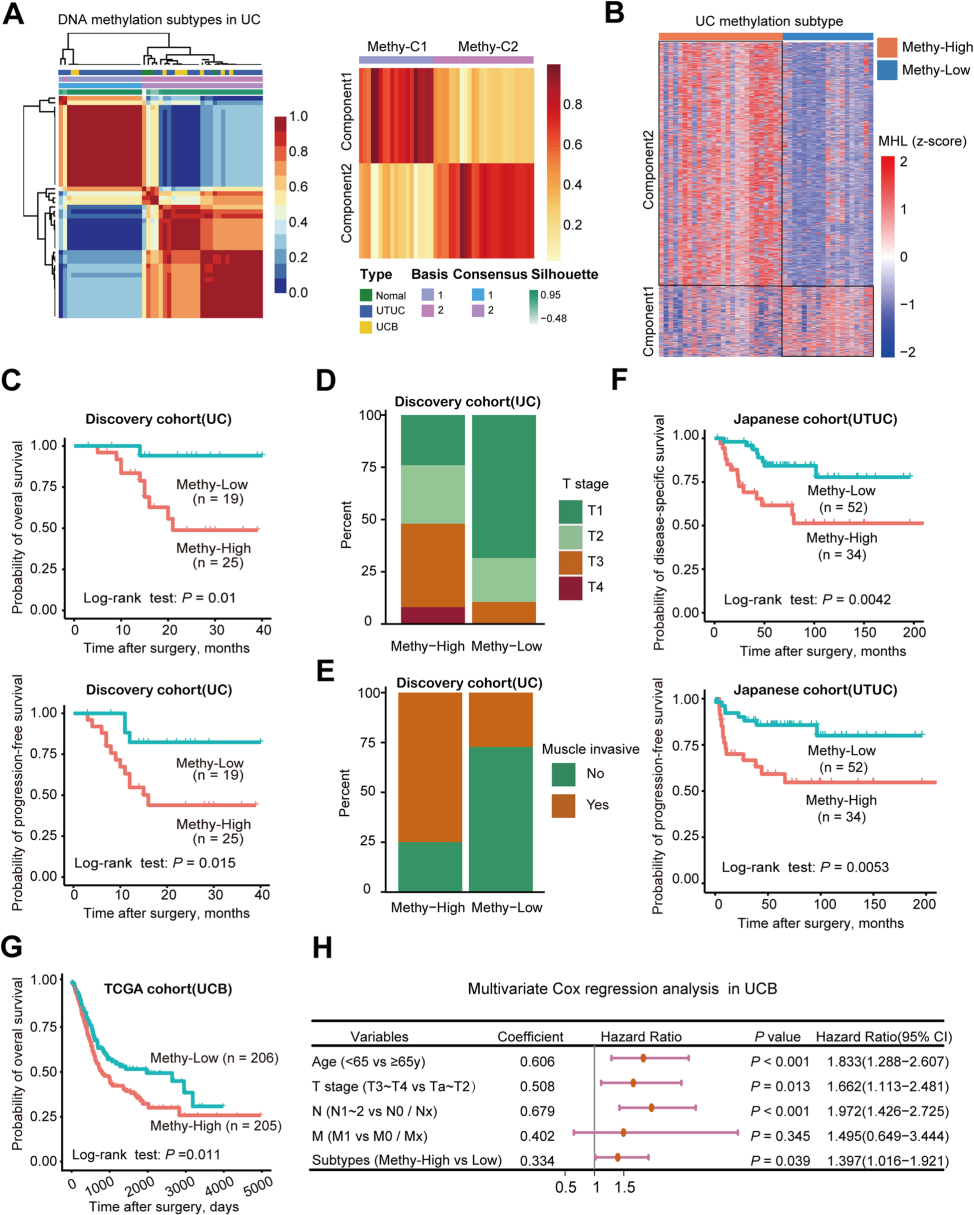
Association between DNA methylation subtype and clinicopathological tumor and patient features. **A** Unsupervised clustering of the top 20,000 most variable DNA methylation haplotype blocks (MHBs) in UC showing two epi-clusters: Methy-C1 (*n*=19; 42.2%) and Methy-C2 (*n*=26; 57.8%). These clusters featured component 1 and component 2 MHBs, respectively. **B** Heatmap of differentially methylated MHBs between the Methy-C1 and Methy-C2 subtypes. Methy-C2 presented frequent hypermethylation compared to Methy-C1, and Methy-C1 was redesignated Methy-Low while Methy-C2 was redesignated Methy-High accordingly. **C** Kaplan–Meier survival curves showing that the DNA methylation subtypes can predict both overall survival and progression-free survival for UC patients. *P*-values were calculated by the log-rank test. *n*, number of cases. **D, E** The bar graph shows the association between the two subtypes of UC and clinicopathologic features. **F** Kaplan–Meier survival curves showed that the DNA methylation subtypes could predict both disease-specific survival and progression-free survival for the Japanese cohort. **G** Kaplan–Meier survival curves showed that the DNA methylation subtypes could predict overall survival for the TCGA muscle-invasive UCB cohort. **H** Forest plots displaying the results of multivariate Cox analysis of demographic variables predicting OS in TCGA UCB patients. CI, confidence interval OS, overall survival

To classify UC samples based on DNA methylation subtypes, we employed the NMF algorithm using the top 20,000 MHBs with the most variable MHLs. We identified two robust components based on 2974 MHBs (Fig. 3A, Additional file 3). Components 1 and 2 contained 659 and 2315 regions, respectively. According to these two basis components, 45 UC samples were classified into two subtypes: Methy-C1 (*n* = 19; 42.2%) and Methy-C2 (*n* = 26; 57.8%). Of these, Methy-C2 presented frequent hypermethylation of 2315 MHBs of component 2, whereas only 659 MHBs of component 1 showed hypermethylation in Methy-C1. As such, we redesignated Methy-C1 as Methy-Low and Methy-C2 as Methy-High accordingly (Fig. 3B, Additional file 4).

### Clinicopathological associations of the methylation subtypes in UC

To examine the clinical relevance of the DNA methylation subtypes in UC, we performed the clinicopathological association assay in 44 UC patients (one sample was missing) from our discovery cohort (Additional file 4), 411 UCB patients from the TCGA cohort, and 86 UTUC patients from the Japanese cohort. We found that patients with tumors belonging to the Methy-High subtype had shorter overall survival than those with tumors belonging to the Methy-Low subtype in the discovery cohort (*P* = 0.01, log-rank test). Likewise, the patients with tumors belonging to the Methy-High subtype also had shorter progression-free survival (*P* = 0.015, log-rank test) (Fig. 3C). In addition, Methy-High samples were enriched for higher tumor stages (≥T3, 12/25 vs. 2/19) and muscle-invasive tumors (19/25 vs. 6/19) compared with Methy-Low samples (Fig. 3D, E). Furthermore, we obtained 475 common regions (Additional file 3) within the top 20,000 most-varied MHBs and EPIC array data from the Japanese cohort. Similarly, we identified two methylation subtypes (Methy-Low (*n* = 52) and Methy-High (*n* = 34)), and it was further confirmed that the Methy-High subtype had shorter disease-specific survival (*P* = 0.0042, log-rank test) and progression-free survival (*P* = 0.0053, log-rank test) (Fig. 3F, Additional file 4).

Since we only determined the WGBS profile of a limited number of UCB patients, we integrated HumanMethylation450 (EPIC) BeadChip array data from TCGA (*n* = 411). We found 601 overlapping MHBs (Additional file 3) out of the top 20,000 most-varied MHBs. Using a similar strategy, we also defined Methy-High and Methy-Low subtypes of patients with UCB (Additional file 1: Fig. S3A and S3B). Strikingly, we found that Methy-High-subtype patients also had shorter overall survival and higher tumor stage than Methy-Low-subtype patients in the UCB group (Fig. 3G and Additional file 1: Fig. S3C, Additional file 4). Almost all TCGA samples were muscle-invasive, so we checked the tumor stage of the samples and found that the Methy-High subtype contained a high proportion of T3–T4 stage samples (Additional file 1: Fig. S3C). T stage, age, lymph node metastasis, distal metastasis, and methylation subtype were found to be significantly correlated with patient survival by univariate Cox regression (Additional file 1: Table S1). Further multivariate Cox regression showed that methylation subtype was an independent prognostic factor (Fig. 3H)

### Methy-High UCs have predominantly basal expression patterns with high immune and stromal scores

**Fig. 4:**
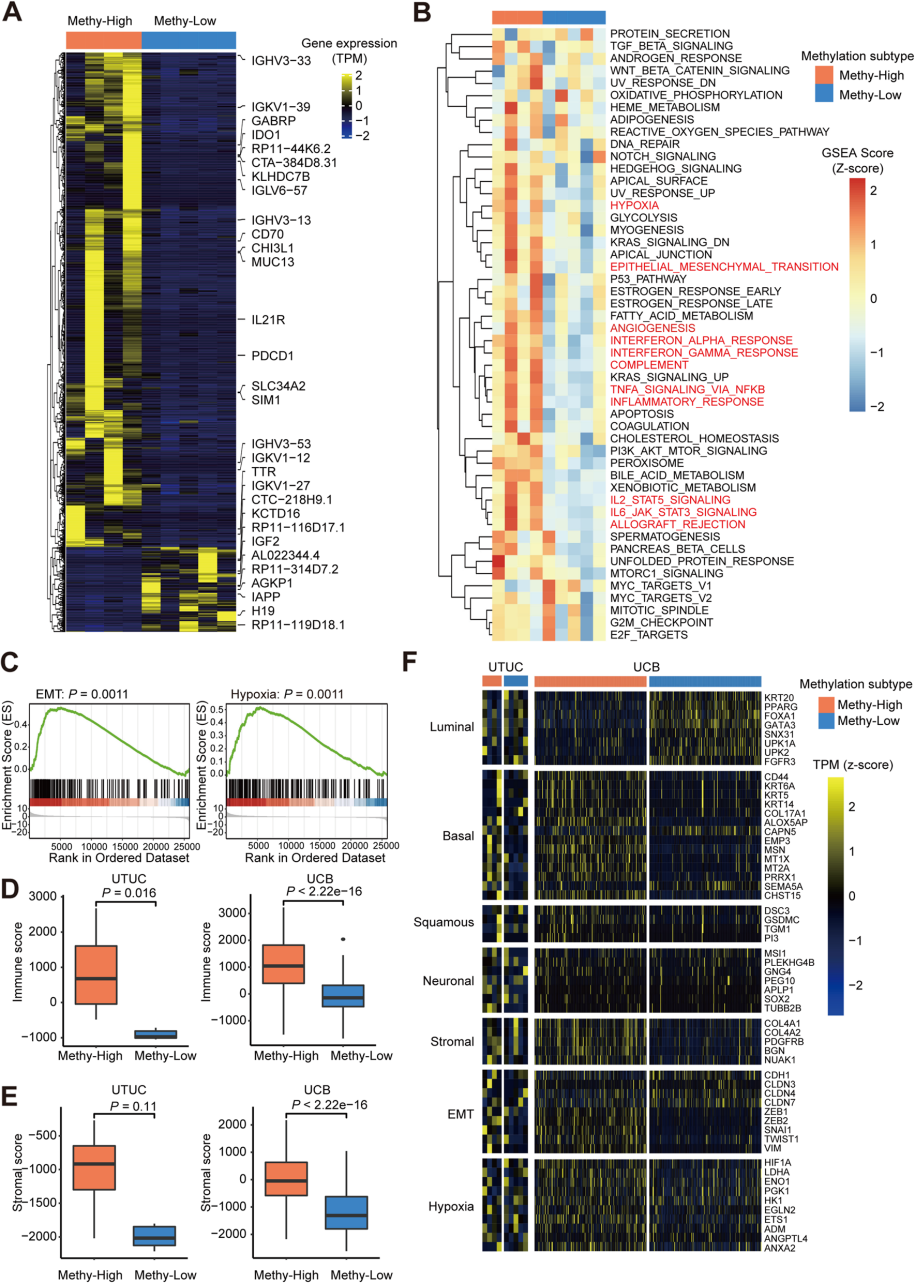
Methy-High UCs predominantly exhibit a basal expression pattern with high immune and stromal scores. **A** Heatmap of differentially expressed genes between Methy-High (*n* = 4) and Methy-Low (*n* = 5) UTUC patients. The top 30 differentially expressed genes are highlighted. **B** Differences in 50 hallmark pathway activities scored with GSVA software between Methy-High and Methy-Low UTUC patients. The *t* values calculated by a linear model are shown. **C** GSEA showing that EMT and hypoxia signaling are enriched in Methy-High UTUC patients compared to Methy-Low UTUC patients. **D, E** Immune and stromal gene expression scores in Methy-High and Methy-Low UC samples. *P*-values were calculated by two-tailed Student’s t test. **F** Expression profiles of the indicated gene pathways of biological relevance implicated in the TCGA UCB cohort in Methy-High and Methy-Low UC samples. EMT, epithelial-mesenchymal transition

To characterize the gene expression profiles that defined the UC methylation subtypes, we generated an RNA-seq dataset from 9 UTUC tumors (Additional file 2), including 4 Methy-High subtype and 5 Methy-Low subtype, and the published TCGA UCB cohort. First, we identified 1664 upregulated and 575 downregulated genes in the Methy-High subtype compared to the Methy-Low subtype (Fig. 4A). We found that the upregulated genes in Methy-High patients were enriched in multiple immune-related pathway terms, such as adaptive immune response, lymphocyte-mediated immunity, and immune receptor (Additional file 1: Fig. S4A). Further enrichment analysis revealed that the immune response, epithelial-mesenchymal transition (EMT), and multiple signaling pathways (hypoxia, glycolysis, and angiogenesis) were upregulated in Methy-High tumors (Fig. 4B, C). Although the sample size was small, Methy-High tumors tended to show higher immune and stromal scores than Methy-Low tumors (*P* = 0.016 in UTUC and *P* < 2.22e-16 in UCB for immune scores; *P* = 0.11 in UTUC and *P* < 2.22e−16 in UCB for stromal scores; Fig. 4D, E).

To better understand the features of each methylation subtype, we compared gene expression between Methy-High and Methy-Low of UTUC and UCB with respect to a set of functional pathways implicated in unique UCB subtypes using the TCGA UCB dataset. We found that Methy-High UC tumors were characterized by upregulation of “basal” markers. Stromal markers, squamous markers, EMT markers, and angiogenesis markers were also upregulated in the Methy-High subtype compared to the Methy-Low subtype (Fig. 4F).

### Methy-High UCs have a T cell inflamed and immunosuppressive environment

**Fig. 5:**
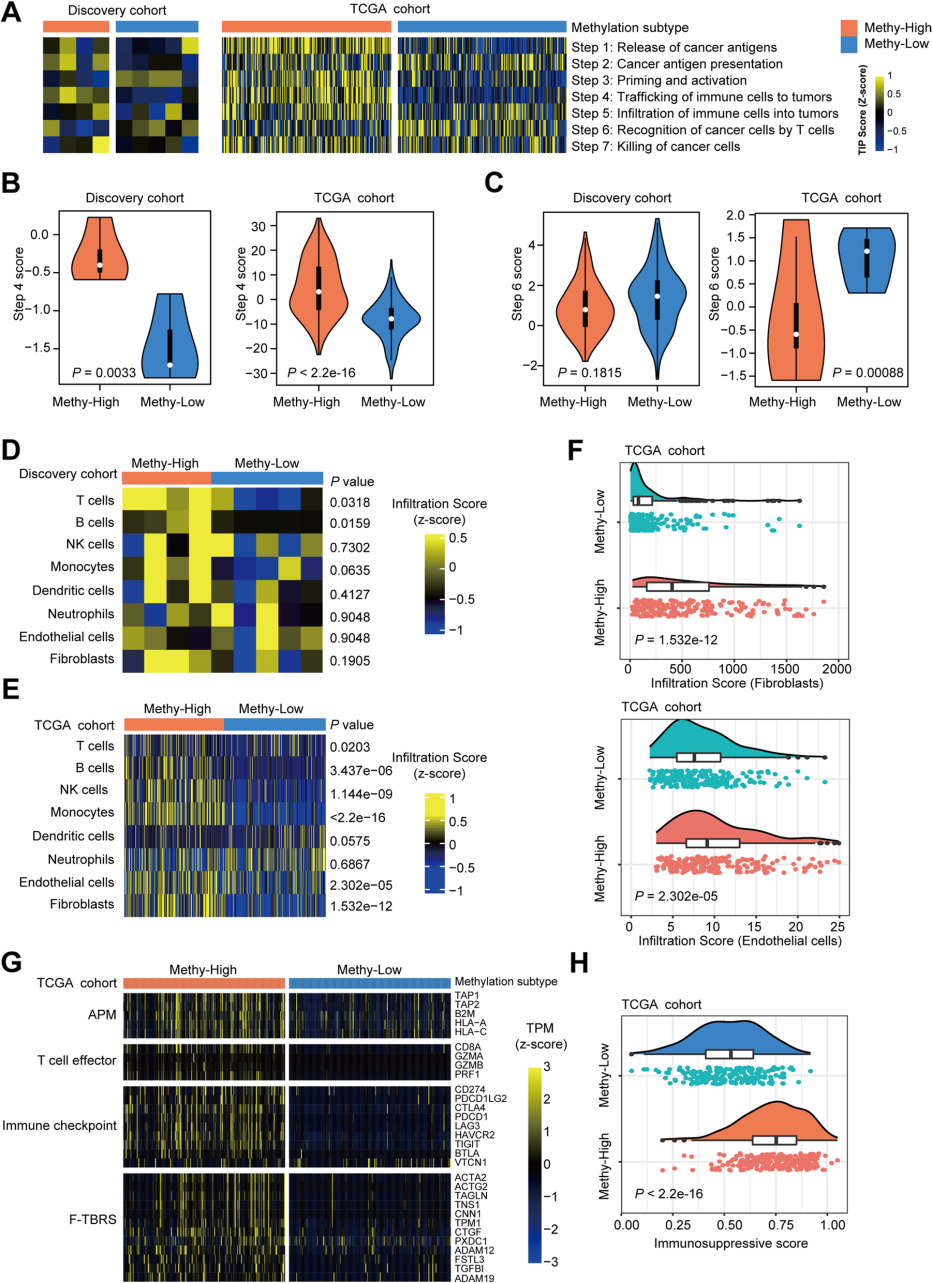
Methy-High UCs have a T cell inflamed and immunosuppressive environment. **A** The heatmap shows the anticancer immunity activity scores in seven steps across the cancer immunity cycle in UC patients with different methylation subtypes. The activity scores were calculated by TIP software. **B, C** Violin diagram analyzing the activity status of Step 4 and Step 6. Step 4: trafficking of immune cells to tumors; Step 6: recognition of cancer cells by T cells. *P*-values were calculated by the Wilcoxon rank test. **D, E, F** Heatmap displaying the population abundance of tissue-infiltrating immune and stromal cell populations in UC patients with different methylation subtypes. The proportion of infiltrating cells was evaluated by MCPcounter. *P*-values were calculated by the Wilcoxon rank test. **G** Expression profiles indicating differences in the anticancer immune response of the two methylation subtypes. APM, antigen presentation machinery, F-TBRS, fibroblast TGFβ response signature. **H** Immunosuppressive scores in Methy-High and Methy-Low UCB samples. *P*-values were calculated by two-tailed Student’s *t* test

We systematically evaluated the immunophenotype of each methylation subtype using TIP [14] and found that cancer antigen presentation and trafficking of immune cells to tumors were enhanced while recognition of cancer cells by T cells was depleted in Methy-High subtype UC patients compared to Methy-Low subtype UC patients (Fig. 5A). Consistently, the score for trafficking of immune cells to tumors was significantly higher in Methy-High patients in both the UTUC and UCB groups (Fig. 5B). However, the score for recognition of cancer cells by T cells was lower in Methy-High patients in both the UTUC and UCB groups (Fig. 5C). Overall, these results indicated that Methy-High patients had inflamed tumors with an immunosuppressive phenotype.

To better evaluate the involvement of immune cells and stromal cells in regulating the immunophenotype of each methylation subtype, we further estimated the abundance of tumor-infiltrating immune cells and stromal cells by MCPcounter [15]. We found that the numbers of tumor-infiltrating T cells, B cells, and monocytes were significantly higher in the Methy-High subtype than in the Methy-Low subtype (Fig. 5D, E). Both fibroblasts and endothelial cells were significantly higher in Methy-High UCB patients than in Methy-Low UCB patients, and a similar trend was also observed in UTUC patients. This may be due to the limited number of patients, and statistical significance was not achieved in UTUC patients (Fig. 5D, E). We further confirmed that the proportions of infiltrating fibroblasts and endothelial cells were higher in Methy-High patients than in Methy-Low patients (Fig. 5F). These results further indicated high involvement of immune cells and stromal components in shaping the immunosuppressive phenotype of Methy-High UC patients. In agreement with these immune response features and stromal reactions, prominently increased expression of proteins related to the antigen presentation machinery and cytolytic activity and the immune evasion markers PD1, PD-L1, PD-L2, and CTLA4 were noted in the Methy-High subtype (Fig. 5G). Furthermore, the Methy-High subtype showed enhanced expression of genes of the pan-fibroblast transforming growth factor b (TGFβ) response signature (F-TBRS) (Fig. 5G), which is an indicator of TGFβ pathway activity in fibroblasts and is implicated in resistance to immunotherapy [16]. These findings suggested that activated stromal signaling potentially plays a prominent role in the immunosuppressive phenotype in Methy-High tumors. Consistently, we further confirmed that the immunosuppressive score was higher in Methy-High patients than in Methy-Low patients (Fig. 5H). Collectively, these results suggest that a combination of immunotherapy and anti-TGFβ treatments might improve the clinical outcome of Methy-High patients.

### Treatment of T24 and UMUC-3 cells with guadecitabine (SGI-110), a DNA methyltransferase inhibitor, showed therapeutic effects

**Fig. 6:**
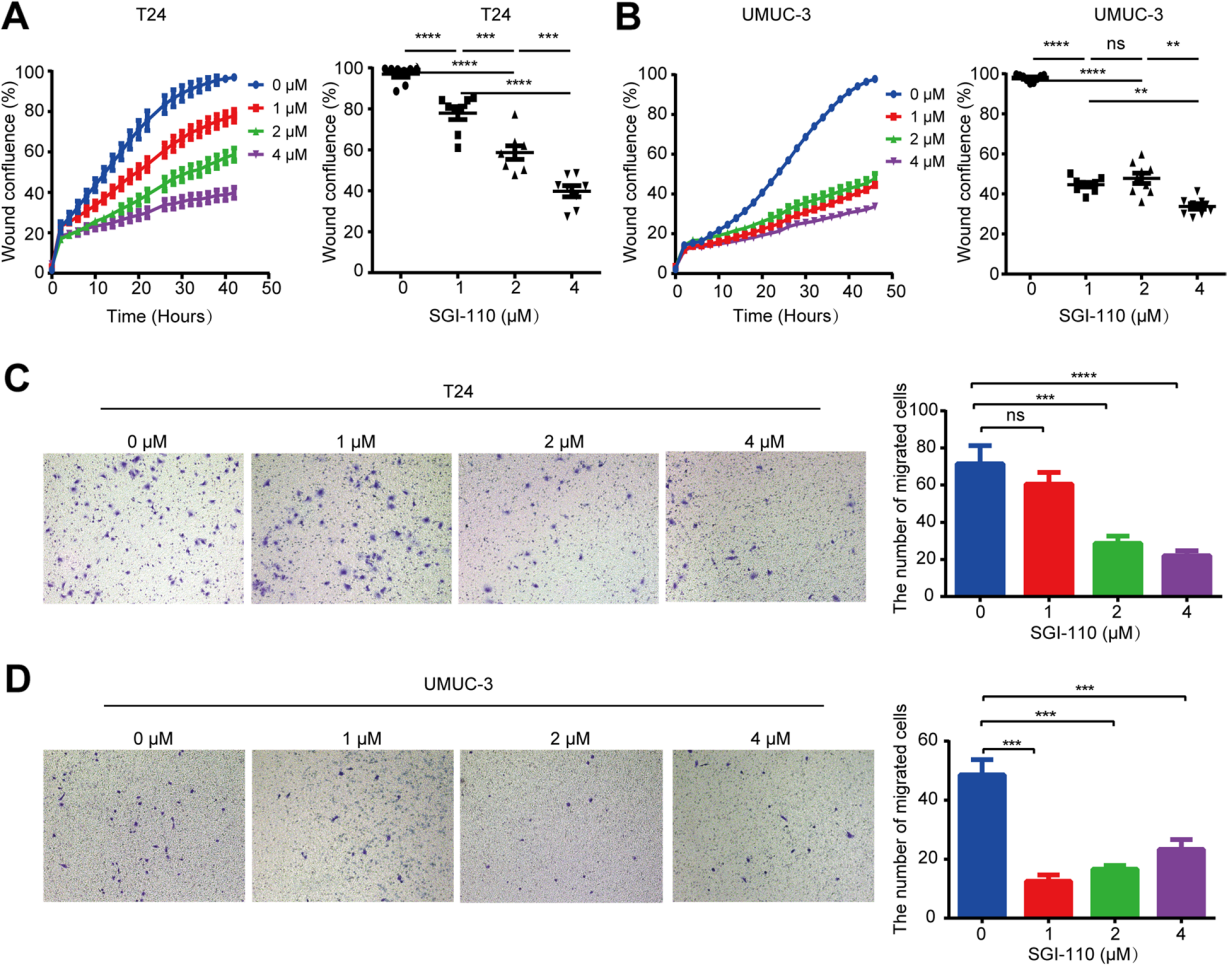
Guadecitabine (SGI-110), a DNA methyltransferase inhibitor, showed therapeutic effects in T24 and UMUC-3 cells. **A, B** Cell migration capacity with or without SGI-110 treatment was evaluated through wound healing assays. Values are the mean ± SD of eight independent experiments. **C, D** Cell invasion capacity with or without SGI-110 treatment was evaluated through Transwell assays. The bar chart on the right represents the number of cells passing through the compartment. Data are presented as the means ± SEM. *n* = 3. *P < 0.05, **P < 0.01, ***P < 0.001, ****P < 0.0001

DNA methyltransferase inhibitors (DNMTis) have emerged as a new therapeutic tool to reverse DNA hypermethylation and associated gene silencing. Therefore, we explored whether DNMTis have efficacy in the treatment of UC. We treated T24 and UMUC-3 cells with SGI-110 [17], a second-generation DNMTi that exhibits a longer half-life and increased exposure. Because DNMTi incorporation into genomic DNA is dependent on cell doubling [18, 19], each cell line was treated with three consecutive 24 h doses (72 h total) of 1 μM, 2 μM, and 4 μM SGI-110 to cover at least one cell doubling cycle before drug removal. Cells were then cultured in drug-free medium, and cell migration and invasion capacity were evaluated by wound healing and transwell assays, respectively. We found that SGI-110 treatment significantly inhibited cell migration (Fig. 6A, B) and invasion capacities (Fig. 6C, D) in both cell lines. UMUC-3 cells were more sensitive to SGI-110 treatment than T24 cells (Fig. 6). Overall, we observed inhibition of cell migration and invasion after withdrawing of SGI-110 treatment, which may explain the antitumor effects observed as a consequence of SGI-110 treatment.

We then monitored DNA methylation changes in T24 and UMUC-3 cells after three consecutive 24 h doses (72 h total) of 2 μM SGI-110 using WGBS (Additional file 2). We observed global DNA demethylation in both cell lines, including demethylation of gene-related regions and intergenic regions (Additional file 1: Fig. S5A). Extensive DNA demethylation was observed at day 3 after treatment with SGI-110 (Additional file 1: Fig. S5B), supporting that the current dose of SGI-110 is effective as a DNA methyltransferase inhibitor. The traditional view is that DNMTi treatment reverses DNA hypermethylation in the promoters of tumor suppressor genes. Recent studies have suggested that both gene body DNA demethylation and repeat demethylation may contribute to the clinical efficacy of DNMTis [19–21]. Consistent with the WGBS data, we confirmed that DNA demethylation occurred in both promoters and/or gene bodies in both cell lines (Additional file 1: Fig. S5C and S5D). Meanwhile, extensive DNA demethylation was identified in all examined repeats, including long interspersed elements (LINEs), SINEs, and long terminal repeats (LTRs) (Additional file 1: Fig. S5E).

To further explore the potential clinical use of DNMTi-based therapies in UC patients, we determined whether the T24 and UMUC-3 cell DNA methylation profiles were comparable to those of primary tumors. Interestingly, both T24 and UMUC-3 cells were clustered within Methy-High subtype tumors (Additional file 1: Fig. S6). Interestingly, the global pattern of DNA methylation was changed after SGI-110 treatment in both cell lines, and this change was more evident in UMUC-3 cells than in T24 cells (Additional file 1: Fig. S5F). The antitumor activities of DNMTis may be specific for different DNA methylation subtypes since DNA methylation profiles are different between DNA methylation subtypes. Considering that both UTUC and UCB have similar methylation profiles, the similar treatment strategy may be applied to both diseases and the efficacy would correlate with methylation subtypes.

### Integrative analysis identifies SGI-110 target genes in T24 and UMUC-3 cells

**Fig. 7:**
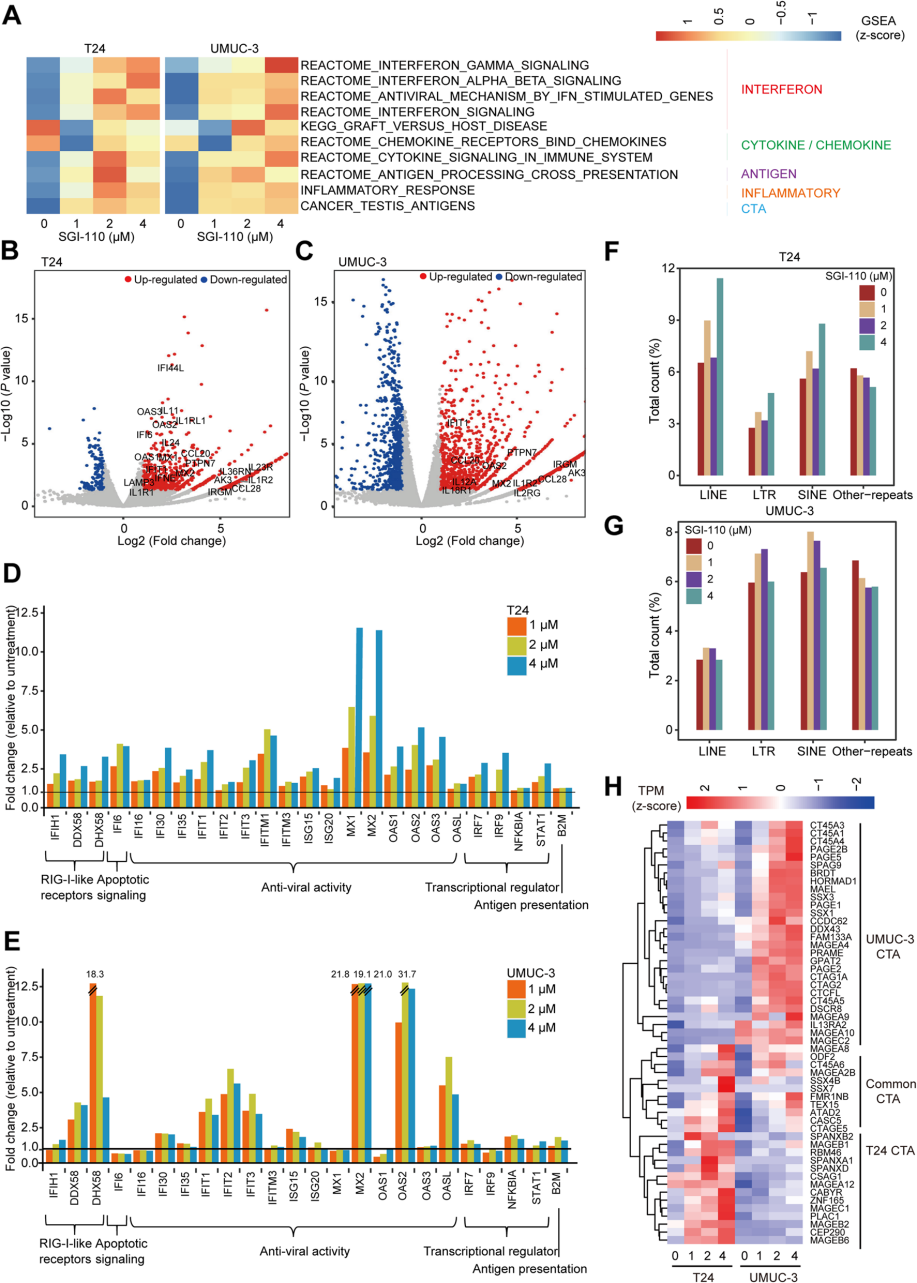
Integrative analysis identifies SGI-110 target genes in T24 and UMUC-3 cells. **A** Summary of GSEA immune-related gene sets upregulated by SGI-110 in T24 and UMUC-3 cells. The “immune” sector is broken down further into specific pathways characterized as part of the interferon response, cytokines/chemokines, antigen presentation, inflammatory, and cancer testis antigen (CTA) categories. **B, C** Volcano plot of gene expression data obtained by RNA-seq in SGI-110-treated T24 and UMUC-3 cells compared to untreated cells. SGI-110 upregulated cytokine/chemokine genes are highlighted. **D, E** Changes in the expression of dsRNA defense genes after treatment with SGI-110. **F, G** The proportions of the read counts from endogenous retroviral long terminal repeats (LTRs), long interspersed nuclear elements (LINEs), and short interspersed nuclear elements (SINEs). **H** Heatmap of cancer testis antigen (CTA) expression in SGI-110-treated T24 and UMUC-3 cells compared to untreated cells. Gene expression was calculated as transcripts per million (TPM) values

To identify the potential therapeutic value of SGI-110, we measured gene expression profiles after treatment with SGI-110 in T24 and UMUC-3 cells (Additional file 2). We found that the upregulated genes after SGI-110 treatment were significantly enriched in multiple antitumor immune pathway terms, such as interferon signaling, cytokine/chemokine, antigen processing, and cancer testis antigens (Fig. 7A). Many genes upregulated by SGI-110 treatment are part of or downstream of antitumor interferon response signaling, including those related to antigen presentation and cytokines/chemokines (Fig. 7B, C). Consistent with previous findings that virus defense-related genes can be upregulated by 5-aza-CdR treatment [20, 21], we found that multiple genes involved in antiviral activity were upregulated in both cell lines after SGI-110 treatment (Fig. 7D, E). This response has been called “viral mimicry” because the cell responds as it would to an exogenous viral infection. Consistently, we identified a larger number of reads from transposon elements, including SINEs, LINEs, and LTRs, after SGI-110 treatment (Fig. 7F, G). These findings were consistent with earlier reports that not only AZA-induced evolutionarily young LTRs but also LINEs and SINEs may potentially trigger activation of the innate immune system in responders to AZA treatment [20, 21]. Finally, we also examined the gene expression levels of cancer testis antigens, which have also been suggested to play important roles in the activation of the immune system and in killing cancer cells [22–24]. Indeed, we observed upregulation of both shared and cell type-specific cancer testis antigens in T24 and UMUC-3 cells (Fig. 7H). Collectively, SGI-110 upregulated multiple antitumor immune pathways.

## Discussion

In this study, we provide comprehensive DNA methylation profiles of UTUC and UCB. Overall, UTUC and UCB showed very similar DNA methylation landscapes. Considering the smaller UCB sample size, we further validated using public cohorts, and the results were as expected (Additional file 1: Fig. S7A). There seems to be a common epigenetic mechanism of positive selection in urothelial carcinogenesis, indicating a potential opportunity for similar management strategies for UC. Of note, by supervised analysis, we also identified differentially methylated regions between UTUC and UCB. Promoter hypermethylated genes in UTUC were enriched in the signaling pathways (Additional file 1: Fig. S7B) that were crucial for cancer development, such as regulation of tumor necrosis factor production (TNF) and T cell activation. Consistently, it is reported that the genes of the TNF pathway were lower expressed in UTUC compared to UCB [25] and the majority of UTUC has a T cell depleted immune contexture [26]. We also found that promoter regions of some EMT-related genes were more hypomethylated in UTUC, such as *ZEB1* and *SLC38A1* (Additional file 1: Fig. S7C&D)*.* These results may partially explain the aggressive clinical behavior and a more advanced presentation of UTUC. Several previous publications have established a prognostic influence of methylation in UC [27, 28], and we found that the methylation levels of some CpG sites were consistent in high-risk patients and in the Methy-High subgroup of UC patients (Additional file 1: Fig. S7E). Notably, with a sufficient number of samples, we identified that DNA methylation subtypes were an independent prognostic marker for muscle-invasive UCB patients (Fig. 3H). The prognostic value of pathological factors for UCs has been discussed elsewhere, and there is a high prevalence of patients with TaT1 due to their often long-term survival and lower risk of cancer-specific mortality compared to patients with T2-4 tumors [29]. More research is needed to determine the role of DNA methylation classification in improving the predictive accuracy of currently available risk tables [30, 31]. Further prospective studies including a larger number of UC patients will be required to validate the prognostic capacity of DNA methylation subtype classification.

Mechanistically, we identified that Methy-High UCs have a predominantly basal expression pattern with high immune and stromal scores and are linked to poor prognosis. This classification, if validated, might be used in the future to predict outcomes of resected UC patients and help identify those who may benefit from chemotherapy. Cisplatin-based combination chemotherapy is the standard-of-care therapy for UC, both as radical perioperative treatment and as palliative first-line treatment for advanced disease [32–34]. Notably, preclinical data indicate that DNMTis will circumvent cisplatin resistance in various cancers, including UCs [35, 36]. Recently, phase I trials of SGI-110 combined with cisplatin and gemcitabine for solid malignancies, including urothelial carcinoma (SPIRE), have been performed, and the addition of SGI-110 to gemcitabine and cisplatin was tolerable, despite some additional myelosuppression, and such combinations warrant further investigation to assess efficacy [37].

More importantly, we identified that Methy-High UCs had a T cell inflamed and immunosuppressive environment, showing therapeutically efficacy to SGI-110 through enhancing antitumor immunity. These findings were consistent with recent investigations suggesting that upregulation of CTAs, activation of endogenous retroviral elements (ERVs), and an antiviral defense response contribute to the clinical efficacy of DNMTis [38, 39]. The 2021 updated European Association of Urology guidelines recommend that metastatic UC patients positive for programmed death ligand 1 (PD-L1) and ineligible for cisplatin receive immunotherapy (atezolizumab or pembrolizumab) [40]. Since the DNA methylation profile is cancer-specific and varies greatly between cancer types, the antitumor activities of DNMTis may be specific for individual cancer types. Our integrative analysis successfully linked the antitumor effects of SGI-110 to detailed epigenetic alterations in UC cells, identified potential therapeutic targets, and provided a rationale for SGI-110 combination with immune checkpoint therapies. Further confirmation of our findings in clinical trials of combination therapy is warranted, with the goal of future implementation in clinical practice.

## Conclusions

Taken together, our results show similar underlying DNA methylation mechanisms in UTUC and UCB, supporting a potential opportunity for similar management strategies for UC. We also provide a roadmap for clinical practice: DNA methylation subtype classification can be used to predict the outcomes of resected UC patients and help identify those who may benefit from cisplatin-based chemotherapy or resensitize cisplatin-resistant cancer cells. Moreover, our results also provide rationale for SGI-110 combination with immune checkpoint therapies in UC. Overall, our findings contribute to the understanding of the pathophysiology of UC and provide prognostic assessment strategies and more individualized treatment recommendations for different UC subtypes.

## Supplementary Information

**Additional file 1: Fig. S1.** Dissection of adjacent urothelium and DNA methylation within 10-kb bins in UC samples. **Fig. S2.** Hypomethylated MHBs are predominant feature in UC. **Fig. S3.** Association between DNA methylation subtype and clinicopathological characteristic, and DNA methylation subtype classification in TCGA cohort. **Fig. S4.** Functional enrichment of differentially expressed genes in methylation subtypes. **Fig. S5.** Transient SGI-110 treatment of T24 and UMUC-3 cells showed extensively DNA demethylation. **Fig. S6.** Unsupervised clustering of cell lines and patients. **Fig. S7.** DNA methylation comparison between UTUC and UCB. **Table S1.** Univariate Cox regression analysis predicting overall survival for patients with methylation subtypes in TCGA.

**Additional file 2.** Summary of sequencing information in samples.

**Additional file 3.** Summary of the regions of methylation classification in three cohorts.

**Additional file 4.** Summary of clinical information in three cohorts.

## Data Availability

The whole-genome bisulfite sequencing data and RNA sequencing data have been deposited in the genome sequence archive of the Beijing Institute of Genomics, National Center for Bioinformation, Chinese Academy of Sciences. The accession numbers for the sequencing data reported in this paper are HRA001562 [41] and HRA001563 [42] at https://ngdc.cncb.ac.cn/gsa/. For the TCGA-BLCA cohort, Methylation450k data and preprocessed bulk RNA-seq data were obtained from UCSC Xena at https://xenabrowser.net/datapages/. For the Japan-UTUC cohort, HumanMethylation850 (EPIC) BeadChip data was obtained from EGAD00010002096 [42] in the European Genome Phenome Archive (http://www.ebi.ac.uk/ega/).

## Abbreviations

DNMTis: DNA methyltransferase inhibitors
EMT: Epithelial-mesenchymal transition
ERVs: Endogenous retroviral elements
F-TBRS: Fibroblast transforming growth factor beta response signature
LINEs: Long interspersed elements
LTRs: Long terminal repeats
MHB: Methylation haplotype block
MHL: Methylation haplotype load
PD-L1: Programmed death ligand 1
SINEs: Short interspersed elements
TGFβ: Transforming growth factor beta
TIP: Tumor immunophenotype profiling
TNF: Tumor necrosis factor
TPM: Transcripts per million transcripts
UC: Urothelial carcinoma
UCB: UC of the bladder
UTUC: Upper tract urothelial carcinoma
