# Supplementary material for: DNA methylation subtypes guiding prognostic assessment and linking to responses the DNA methyltransferase inhibitor SGI-110 in urothelial carcinoma
Source: BMC Med. 2022 Jul 18;20:222. doi: 10.1186/s12916-022-02426-w (PMC9290251; doi:10.1186/s12916-022-02426-w)
Supplement: Supplementary file 1 — Additional file 1: Fig. S1. Dissection of adjacent urothelium and DNA methylation within 10-kb bins in UC samples. Fig. S2. Hypomethylated MHBs are predominant feature in UC. Fig. S3. Association between DNA methylation subtype and clinicopathological characteristic, and DNA methylation subtype classification in TCGA cohort. Fig. S4. Functional enrichment of differentially expressed genes in methylation subtypes. Fig. S5. Transient SGI-110 treatment of T24 and UMUC-3 cells showed extensively DNA demethylation. Fig. S6. Unsupervised clustering of cell lines and patients. Fig. S7. DNA methylation comparison between UTUC and UCB. Table S1. Univariate Cox regression analysis predicting overall survival for patients with methylation subtypes in TCGA. [file 12916_2022_2426_MOESM1_ESM.docx]

Supplementary Information for

**DNA methylation subtypes guiding prognostic assessment and linking to responses the DNA methyltransferase inhibitor SGI-110 in urothelial carcinoma**

**Supplementary Figures and Tables**

**Fig. S1** Dissection of adjacent urothelium and DNA methylation within 10-kb bins in UC samples.

**Fig. S2** Hypomethylated MHBs are predominant feature in UC.

**Fig. S3** Association between DNA methylation subtype and clinicopathological characteristic, and DNA methylation subtype classification in TCGA cohort.

**Fig. S4** Functional enrichment of differentially expressed genes in methylation subtypes.

**Fig. S5** Transient SGI-110 treatment of T24 and UMUC-3 cells showed extensively DNA demethylation

**Fig. S6** Unsupervised clustering of cell lines and patients.

**Fig. S7** DNA methylation comparison between UTUC and UCB.

**Table S1.** Univariate Cox regression analysis predicting overall survival for patients with methylation subtypes in TCGA.

**Supplementary Figures:**

**
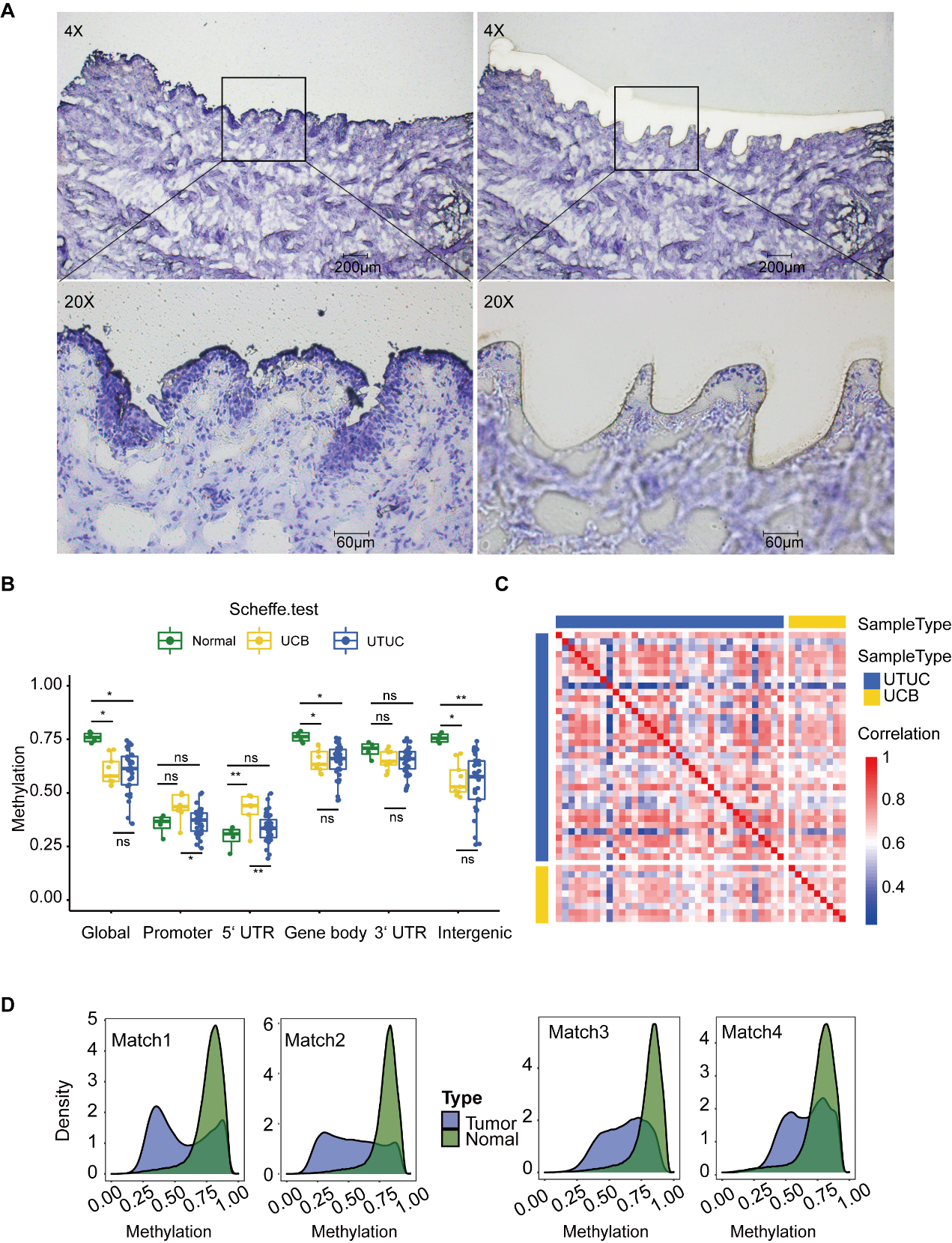
**

**FIGURE S1. Dissection of djacent urothelium and DNA methylation within 10-kb bins in UC samples.**

(A) Field representation of adjacent urothelium collected by CellCut Plus microsystem. (B) Global changes in average 5mC levels in different genomic elements determined by WGBS (the promoter is defined as ±1000 bp of the TSS) in UCB (*n* = 9), UTUC (*n* = 36) and paired adjacent urothelium specimens (*n* = 4) (Scheffe test *P < 0.05, **P < 0.01, ***P < 0.001, ****P < 0.0001)

(C) Correlation of average DNA methylation within all genome-wide 10-kb bins in the tumor of UCB (n = 9) and UTUC (n = 36).

(D) Density maps showing distribution of the methylation levels of all genome-wide 10-kb bins in paired UTUC tumors and adjacent urothelium (n = 4).


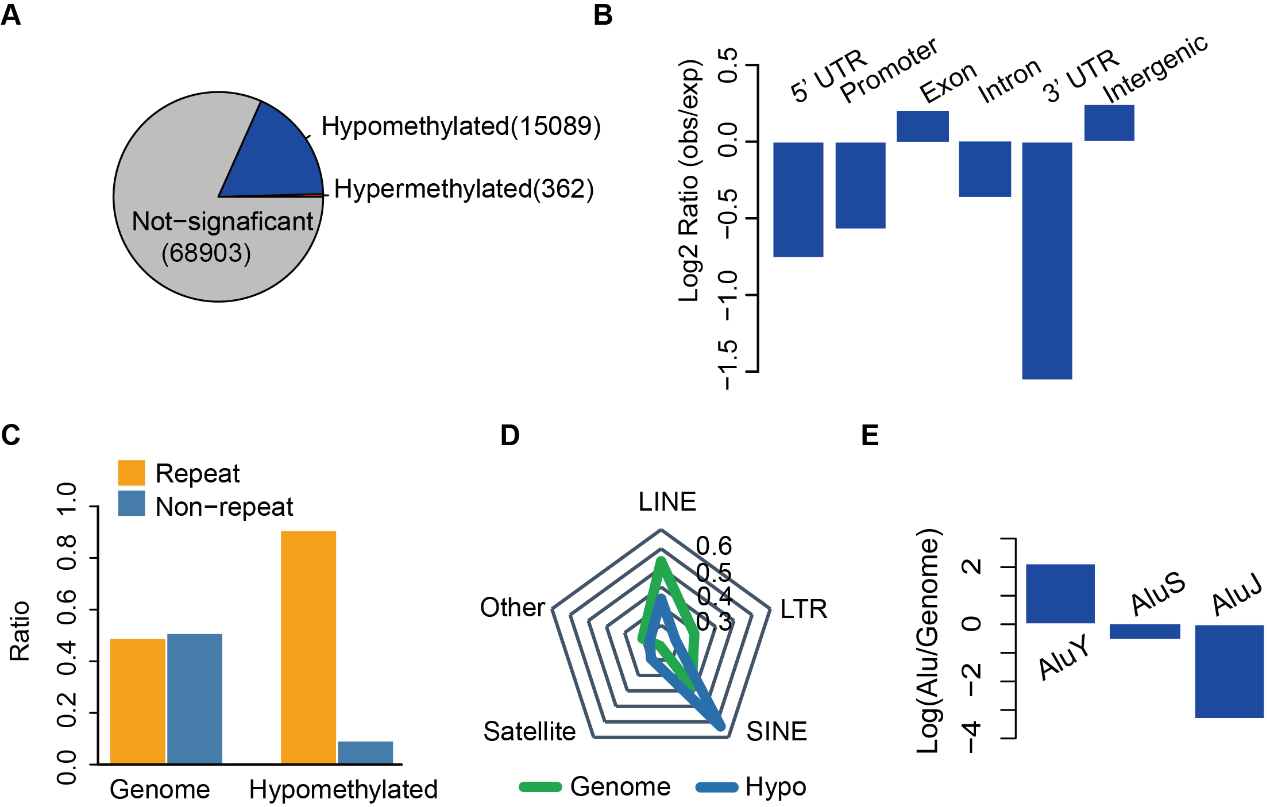


**FIGURE S2. Hypomethylated MHBs are predominant feature in UC.**

(A)Pie chart showing the ratio of hypermethylated and hypomethylated MHBs in UC, compared to adjacent urothelium.

(B)The enrichment (Log2 (obs/exp)) of hypomethylated MHBs in genome elements. (C)The ratio of hypomethylated MHBs located in repeat to non-repeat sequence, with hg19 genome proportions as the background.

(D)The proportion of hypomethylated MHBs in various repeat sequence.

(E)The enrichment (log2 (Alu/Genome)) of hypomethylated MHBs in different evolutionary Alu elements.


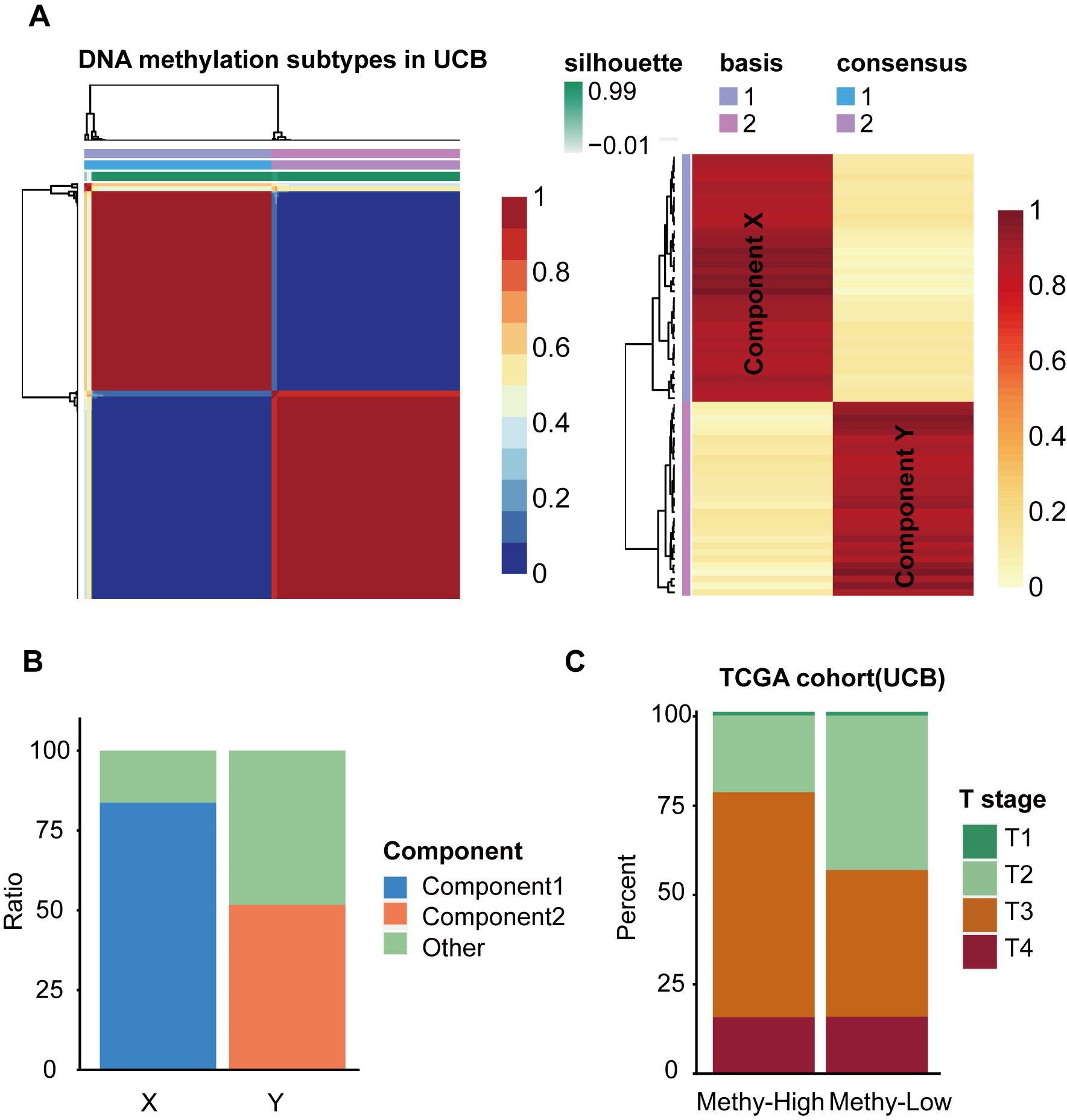


**FIGURE S3. Association between DNA methylation subtype and clinicopathological characteristic in TCGA UCB cohort.**

(A) Unsupervised clustering of overlap 601 DNA methylation haplotype blocks (MHBs) in UCB showing two epi-clusters: Methy-C1 (n=206; 50.1%) and Methy-C2 (n=205; 49.9%), and featured with component X and component Y MHBs, respectively.

(B) The ratio represents the relationship between the regions of the two components (X and Y) from UCB and components that we found.

(C) The bar graph shows the association between the two subtypes of UCB and pathologic stages.


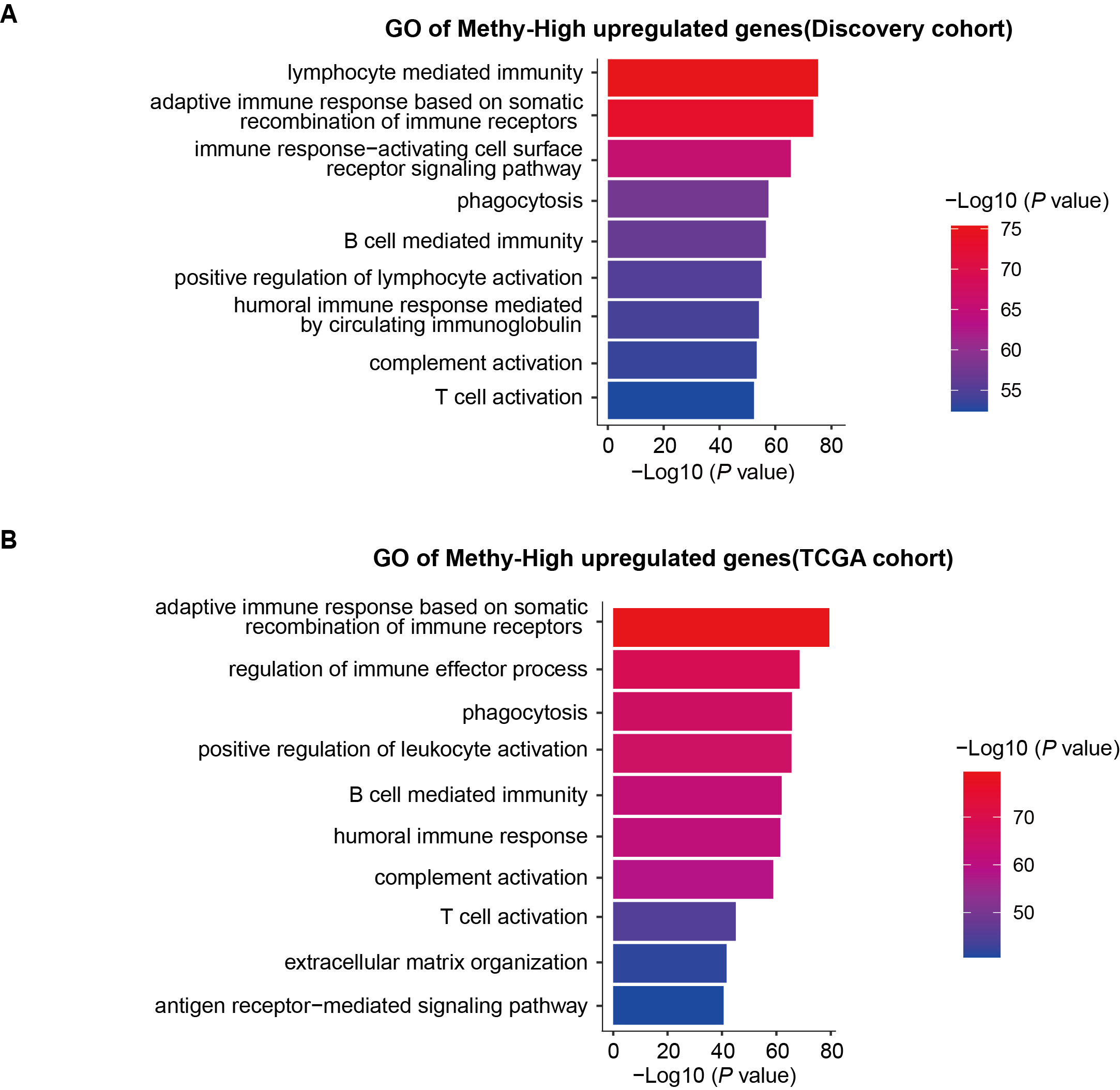


**FIGURE S4. Functional enrichment of differentially expressed genes in methylation subtypes.**

(A-B) GO pathway analysis of the upregulated genes in the Methy-High subtype.


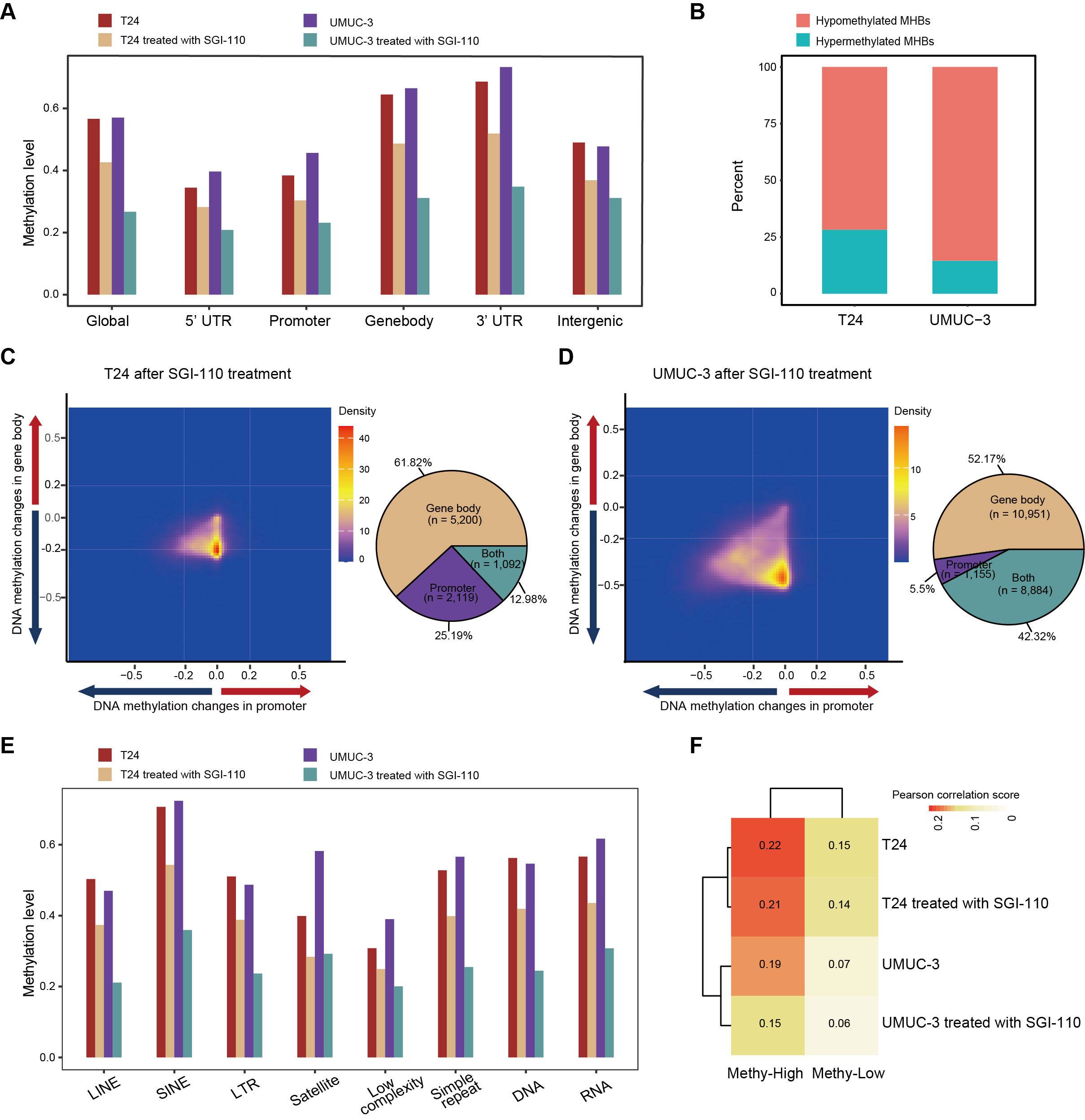


**FIGURE S5.** **Transient SGI-110 treatment of T24 and UMUC-3 cells showed extensively DNA demethylation**

(A) Global changes of average 5mC levels of T24 and UMUC-3 cells after SGI-110 treatment (2μM) in different genomic elements determined by WGBS (promoter is defined as ±1000 bp of TSS).

(B) Box plots represent the proportion of differentially methylated MHBs in T24 and UMUC-3 cells after SGI-110 treatment (2μM).

(C, D) The kernel density scatter plot shows genome-wide DNA methylation changes of promoter and gene body in T24 and UMUC-3 cells after SGI-110 treatment (2μM).

(E) Global changes of average 5mC levels of repeat regions in T24 and UMUC-3 cells after SGI-110 treatment (2μM).

(F) Pearson’s correlation of 5mC patterns of the indicated samples. 5mC levels were measured within a 10-kb bin. The white-red bar indicates the correlation score from low to high.


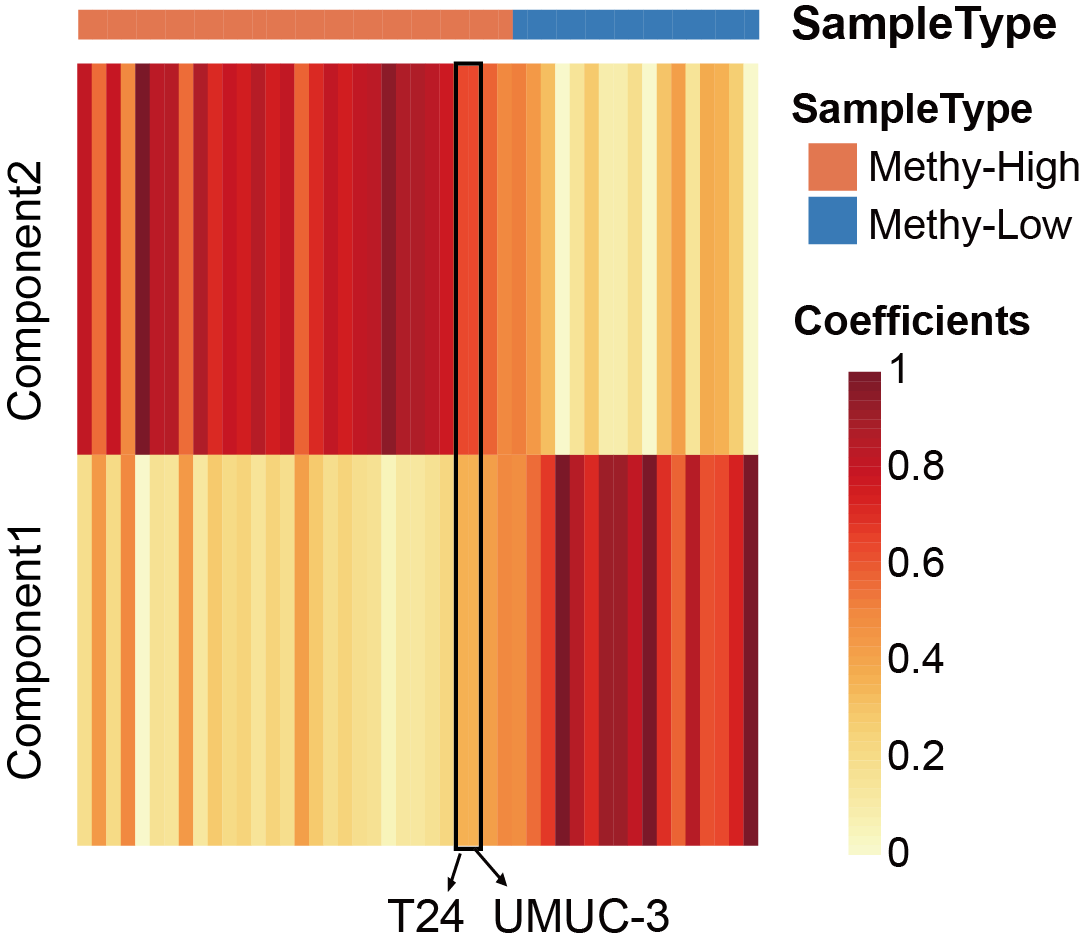


**FIGURE S6. Unsupervised clustering of cell lines and patients.** The weight ratio of cell lines in two components.


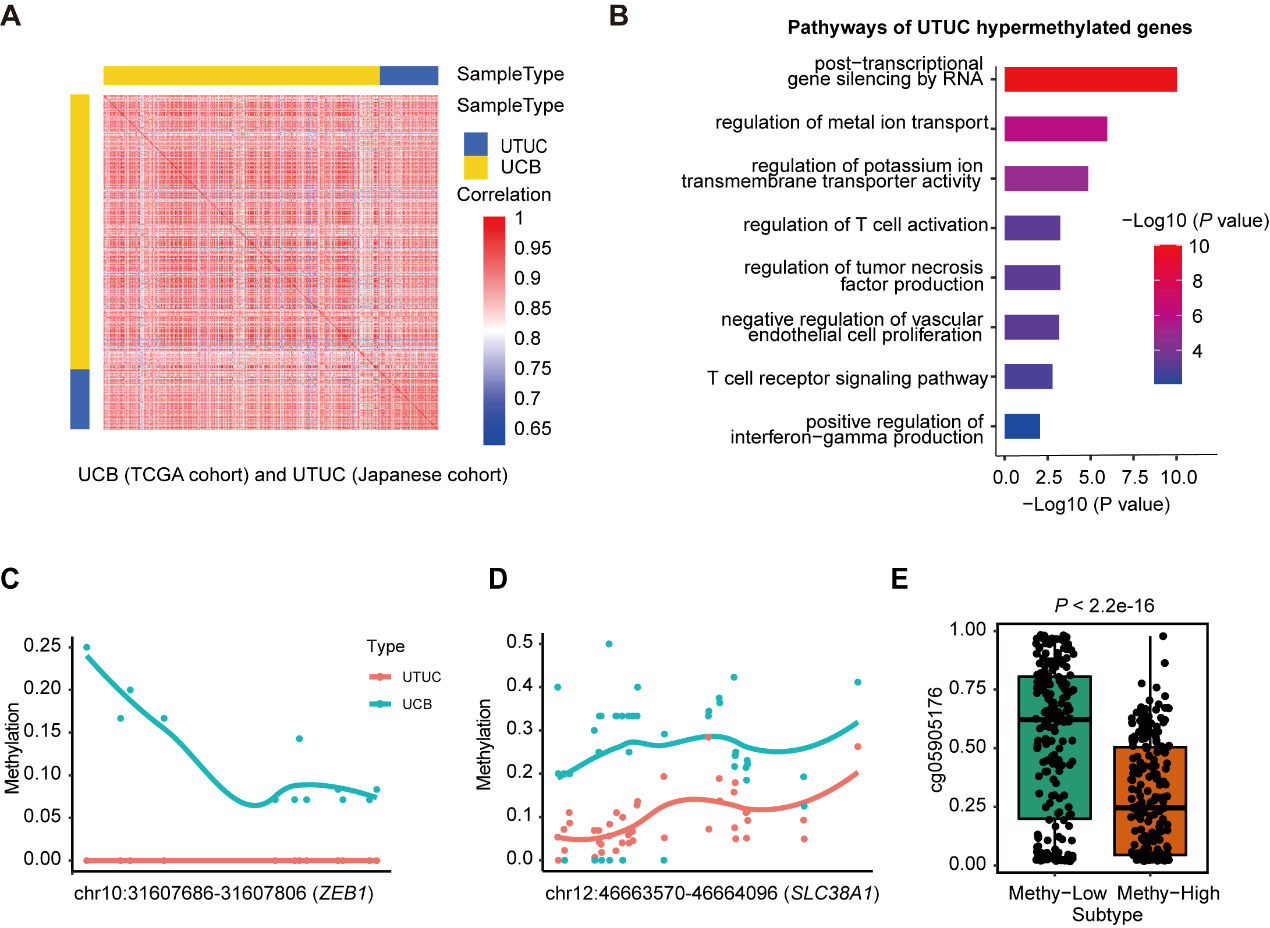


**FIGURE S7. DNA methylation comparison between UTUC and UCB.**

(A)Correlation of average DNA methylation within all genome-wide 10-kb bins in the tumor of UCB (TCGA cohort) and UTUC (Japanese cohort).

(B) Pathway analysis of the hypermethylated genes in UTUC compared to UCB.

(C-D) Methylation levels in promoter regions of EMT-related genes between UTUC and UCB.

(E) Methylation levels of cg05905176 in Methy-High and Methy-Low subtypes.

.

**Table S1. Univariate Cox regression analysis predicting overall survival for patients with methylation subtypes in TCGA**

| Variables | Univariable | | |
| --- | --- | --- | --- |
|  | HR | 95% CI | *P* |
| Gender (Male vs Female) | 1.1196 | 0.8093~1.549 | 0.495 |
| Age (<65 vs ≥65y) | 1.963 | 1.404~2.745 | <0.001 |
| T stage (T3&T4 vs Ta, T1,T2) | 2.207 | 1.519~3.207 | <0.001 |
| N (N1~2 vs N0/Nx) | 2.1553 | 1.602~2.899 | <0.001 |
| M (M1 vs M0/Mx) | 2.673 | 1.311~5.448 | 0.00683 |
| Methylation subtype (Methy-High vs Methy-Low) | 1.464 | 1.088~1.971 | 0.0119 |
